# Characterization of SNP and Structural Variations in the Mitochondrial Genomes of *Tilletia indica* and Its Closely Related Species Formed Basis for a Simple Diagnostic Assay

**DOI:** 10.1371/journal.pone.0166086

**Published:** 2016-11-04

**Authors:** Mui-Keng Tan, Harsh Raman, Grant Chambers, Indu Sharma, Zhiliang Chen, Nandan Deshpande, Marc R. Wilkins

**Affiliations:** 1 Elizabeth Macarthur Agricultural Institute, NSW Department of Primary Industries, Menangle, NSW, Australia; 2 Wagga Wagga Agricultural Research Institute, NSW Department of Primary Industries, Wagga Wagga, NSW, Australia; 3 Department of Plant Breeding, Genetics and Biotechnology, Punjab Agricultural University, Ludhiana, India; 4 School of Biotechnology and Biomolecular Sciences, The University of New South Wales, Kensington, NSW, Australia; 5 Ramaciotti Centre for Genomics, The University of New South Wales, Kensington, NSW, Australia; National University of Ireland - Galway, IRELAND

## Abstract

*Tilletia indica* causes the disease Karnal bunt in wheat. The disease is under international quarantine regulations. Comparative mitochondrial (mt) genome analysis of *T*. *indica* (KX394364 and DQ993184) and *T*. *walkeri* (EF536375) has found 325 to 328 SNPs, 57 to 60 short InDels (from 1 to 13 nt), two InDels (30 and 61 nt) and five (>200 nt) presence/absence variations (PAVs) between the two species. The mt genomes of both species have identical gene order. The numbers of SNPs and InDels between the mt genomes of the two species are approximately nine times of the corresponding numbers between the two *T*. *indica* isolates. There are eight SNPs between *T*. *indica* and *T*. *walkeri* that resulted in amino acid substitutions in the mt genes of *cob*, *nad2* and *nad5*. In contrast, there is no amino acid substitution in the mt genes of the *T*. *indica* isolates from the SNPs found. The five PAVs present in *T*. *indica* (DQ993184) are absent in *T*. *walkeri*. Four PAVs are more than 1 kb and are not present in every *T*. *indica* isolate. Analysis of their presence and absence separates a collection of *T*. *indica* isolates into 11 subgroups. Two PAVs have ORFs for the LAGLIDAG endonuclease and two have ORFs for the GIY-YIG endonuclease family, which are representatives of homing endonuclease genes (HEGs). These intron- encoded HEGs confer intron mobility and account for their fluid distribution in *T*. *indica* isolates. The small PAV of 221 bp, present in every *T*. *indica* isolate and unique to the species, was used as the genetic fingerprint for the successful development of a rapid, highly sensitive and specific loop mediated isothermal amplification (LAMP) assay. The simple procedure of the LAMP assay and the easy detection formats will enable the assay to be automated for high throughput diagnosis.

## Introduction

*T*. *indica* causes the disease, Karnal bunt. It replaces part of the seed with a black powdery mass containing millions of spores and produces a strong unpleasant odour like rotten fish. The pathogen has a negligible effect on yield but the fishy smell has serious consequences for the marketability of wheat. The fungus is thus subjected to very strict quarantine regulations in Australia (http://www.agriculture.gov.au/pests-diseases-weeds/plant/karnal-bunt) where more than half of the wheat production is exported, and also in other countries not known to have the pathogen, particularly the EU countries [1, 2] and China [3]. Risk analysis had indicated that the socio-economic impact of a Karnal bunt incursion from loss of export markets and costs of controlling the establishment and spread of the pathogen is huge in Australia and the EU countries [1, 2, 4, 5, 6]. History has also shown that a Karnal bunt incursion in Arizona in 1996 had resulted in a ban of US wheat imports by 32 countries [7].

*T*. *indica* was first reported in Karnal, India [8, 9] and subsequently found to have established in surrounding areas including India, Afghanistan, Pakistan, Nepal and Iraq [10] and Iran [11]. It is thought to have been introduced from Asia to Mexico where it was first recorded in 1971 [12]. It has since been reported in Brazil [13], the USA [14] and South Africa [15]. Once present, this pathogen is extremely difficult to eradicate.

A detectable level of the disease would indicate the pre-existence of the pathogen for several years [16]. Prompt detection at the incursion stage is very important to prevent disease establishment and the spread of the pathogen in a new area. Not all of the wheat heads (spikes) in a crop are infected and not all the grains in a spike are infected. Thus early detection of Karnal bunt in a standing wheat crop in the field is highly unlikely. The key to its detection is thus the deployment of strategic surveillance and quarantine regulations in the wheat supply chain with the use of very sensitive and accurate diagnostic tools to detect and identify a small number of spores.

The diagnostic protocol for *T*. *indica* in the International Standards for Phytosanitary Measures (ISPM No. 27 Annex 4) [17] endorses a range of identification methods from morphology under the microscope to molecular methods using PCR, both conventional and real-time. Morphological identification requires the expertise of experienced bunt pathologists and is time-consuming and very straining for the eyes during disease surveillance. Some of the PCR protocols listed require the germination of the spores for DNA extraction. Germination of *Tilletia* spores takes at least 2 weeks [18] and this may not occur. The molecular differentiation between *T*. *indica* and its closely related species, *T*. *walkeri* is based on a small ITS sequence region that differs by only one nucleotide [19, 20].

The direct real-time PCR assay on teliospores [21] requires expensive fluorescent probes and instrumentation including thermal cyclers and real-time PCR machines in addition to skilled technical staff proficient in molecular techniques. In times of wide-scale surveillance in an incursion when high throughput is required, the workflow can be cumbersome.

Genetic differentiation of *T*. *indica* from other *Tilletia* species has to date been restricted to only the nuclear ribosomal genes [22], which showed that *T*. *indica* and *T*. *walkeri* are very closely related. *T*. *walkeri* only causes bunt of ryegrass and does not infect wheat, so unequivocal differentiation of this closely related species from *T*. *indica* is critical from a quarantine perspective. Genome sequence data for *T*. *indica* that can be used for development of more efficient and accurate diagnostic assays is limited, which hampers the understanding of genetic variability within *T*. *indica* isolates and between *T*. *indica* and *T*. *walkeri*. We thus undertook next generation sequencing of an isolate of *T*. *indica*. The *de novo* assembly of a genome is time-consuming and challenging but to date we have obtained an accurate sequence (sequencing depth of 5245 times) of the mitochondrial (mt) genome of one isolate of *T*. *indica*.

This paper described the comparative analysis of the mt contig of a *T*. *indica* isolate obtained from next generation sequencing with the reference mt genomes of *T*. *indica* (DQ993184) and *T*. *walkeri* (EF536375) which were obtained by conventional Sanger sequencing of libraries of DNA clones. The genomes comparison enabled the identification of SNPs, short sequence insertions and deletions (InDels) and five presence/absence variations (PAVs). The distribution of the five PAVs was investigated in a collection of *T*. *indica* isolates. The results were discussed with a focus on their application to underpin the successful development of a simple and specific diagnostic assay for *T*. *indica* for disease surveillance and/or quarantine.

## Materials and Methods

### Materials

The DNA samples of the *Tilletia* species used in this study (Table 1) were the same as those published in Tan et al. [21]. The materials included a total of 30 *T*. *indica* isolates from India, four from Mexico, and one each from USA and Pakistan. Other *Tilletia* species included *T*. *walkeri* (3), *T*. *horrida* (1), *T*. *ehrhartae* (6), *T*. *tritici* (4), *T*. *contraversa* (3), *T*. *bromi* (2), *T*. *fusca* (2) and *T*. *laevis* (2).

**Table 1:** *Tilletia* species, their host and geographical origin and their suppliers.

Species	Collection No	Host	Origin/Year	Supplier^a^
*T*. *indica*	Ti 1	*Triticum aestivum*	Sonnora, Mexico	1
*T*. *indica*	Ti 2	*T*. *aestivum*	Sonnora, Mexico	1
*T*. *indica*	Ti 3	*T*. *aestivum*	Sonnora, Mexico	1
*T*. *indica*	Ti 6	*T*. *aestivum*	Pakistan	1
*T*. *indica*	Ti 7	*T*. *aestivum*	Dakka, India	1
*T*. *indica*	Ti 8	*T*. *aestivum*	Ropar, India	1
*T*. *indica*	Ti 9	*T*. *aestivum*	Guerdersmir, India	1
*T*. *indica*	Ti 10	*T*. *aestivum*	California, USA	1
*T*. *indica*	WL1562	*T*. *aestivum*	India	1
*T*. *indica*	P1	*T*. *aestivum*	India	2
*T*. *indica*	P2	*T*. *aestivum*	Amritsar, India	2
*T*. *indica*	P3	*T*. *aestivum*	Ferozepur, India	2
*T*. *indica*	P4	*T*. *aestivum*	Bathinda, India	2
*T*. *indica*	P5	*T*. *aestivum*	Nawanshahar, India	2
*T*. *indica*	P6	*T*. *aestivum*	Faridkot, India	2
*T*. *indica*	P7	*T*. *aestivum*	Sangrur, India	2
*T*. *indica*	P8	*T*. *aestivum*	Mansa, India	2
*T*. *indica*	P9	*T*. *aestivum*	Gurdaspur, India	2
*T*. *indica*	P10	*T*. *aestivum*	Hoshiarpur, India	2
*T*. *indica*	P11	*T*. *aestivum*	Ludhiana, India	2
*T*. *indica*	P12	*T*. *aestivum*	Ropar, India	2
*T*. *indica*	P13	*T*. *aestivum*	Pantnagar, India	2
*T*. *indica*	P14	*T*. *aestivum*	Harayana,India	2
*T*. *indica*	P15	*T*. *aestivum*	Pradesh, India	2
*T*. *indica*	P16	*T*. *aestivum*	Uttar Pradesh, India	2
*T*. *indica*	Ps2	*T*. *aestivum*	Gurdaspur, India	2
*T*. *indica*	Ps6	*T*. *aestivum*	Gurdaspur, India	2
*T*. *indica*	Ps7	*T*. *aestivum*	Gurdaspur, India	2
*T*. *indica*	Ps9	*T*. *aestivum*	Gurdaspur, India	2
*T*. *indica*	Ps12	*T*. *aestivum*	Gurdaspur, India	2
*T*. *indica*	Ps14	*T*. *aestivum*	Gurdaspur, India	2
*T*. *indica*	Ps17	*T*. *aestivum*	Gurdaspur, India	2
*T*. *indica*	Ps21	*T*. *aestivum*	Gurdaspur, India	2
*T*. *indica*	Ps23	*T*. *aestivum*	Gurdaspur, India	2
*T*. *indica*	M8602	*T*. *aestivum*	Mexico	3
*T*. *indica*	Jy01	*T*. *aestivum*	India	3
*T*. *walkeri*	Tw4	*Lolium multiflorum*	Georgia, USA	1
*T*. *walkeri*	DAR16720	*L*. *perenne*	NSW, Australia	4
*T*. *walkeri*	DAR16802	*L*. *perenne*	NSW, Australia	4
*T*. *horrida*	Th2	*Oryza sativa*	California, USA	1
*T*. *ehrhartae*	VPRI32078	*Ehrharta calycina*	SA, Australia	5
*T*. *ehrhartae*	BRIP45365	*Ehrharta calycina*	SA, Australia	6
*T*. *ehrhartae*	BRIP26818	*Ehrharta calycina*	WA, Australia	6
*T*. *ehrhartae*	BRIP28392	*Ehrharta calycina*	SA, Australia	6
*T*. *ehrhartae*	BRIP39762	*Ehrharta calycina*	SA, Australia	6
*T*. *ehrhartae*	BRIP45363	*Ehrharta calycina*	SA, Australia	6
*T*. *tritici* (*T*. *caries*)	S4	*T*. *aestivum*	Sejet, Denmark	7
*T*. *tritici* (*T*. *caries*)	S6	*T*. *aestivum*	Sejet, Denmark	7
*T*. *contraversa*^c^	756	*Triticum* sp.	Idaho, USA	8
*T*. *contraversa*	M973111	*Triticum* sp.	Ontario, Canada	8
*T*. *contraversa*	177	*Triticum* sp.	Utah, USA	8
*T*. *bromi*	64	*Bromus japonicus*	Idaho, USA	8
*T*. *bromi*	120	*B*. *japonicus*	Idaho, USA	8
*T*. *fusca*	314A	*Vulpia microstachys*	Washington, USA	9
*T*. *fusca*	344	*V*. *microstachys*	Washington, USA	9
*T*. *tritici* (*T*. *caries*)	DAR40492	*T*. *aestivum*	NSW, Australia	4
*T*. *tritici* (*T*. *caries*)	DAR34387	*T*. *aestivum*	VIC, Australia	4
*T*. *laevis*	DAR73302	*T*. *aestivum*	NSW, Australia	4
*T*. *laevis*	WW05/0037	*T*. *aestivum*	NSW, Australia	10

^a^ **1**: K. Hughes, Central Science Laboratory, Sand Hutton, York, Y0411LZ, UK; **2**: I. Sharma, Punjab Agricultural University, Ludhiana 141004, Punjab, India; **3**: J. Yi, Shanghai Entry-Exit Inspection and Quarantine Bureau, China; **4**: M. Priest, Orange Agricultural Institute, NSW Dept. of Primary Industries, Orange, NSW 2800, Australia; **5**: R. Jones, Dept. of Primary Industries, Primary Industries Research Victoria, Vic 3156, Australia; **6**: R. G. Shivas, Plant Pathology Hebarium, Dept. of Primary Industries and Fisheries, Indooroopilly, Qld 4068, Australia; **7**: S.K. Christiansen, Plant Research Department, Risø National Laboratory, PO Box 49, DK-4000 Roskilde, Denmark; **8**: J.G. McDonald, Centre for Plant Quarantine Pests, Canadian Food Inspection Agency, Ontario, Canada; **9**: L.M. Carris, Dept. of Plant Pathology, Washington State University, Pullman, USA; **10**: K. Wratten, Wagga Wagga ARI, NSW Dept. of Primary Industries, Wagga Wagga, NSW 2650, Australia.

### Sequencing

The DNA sample sequenced was a total DNA extract from mycelium germinated from a teliospore of the isolate, Ps2 [21] using the method as described in [17]. Whole-genome sequencing of the DNA sample was performed at Ramaciotti Centre for Genomics, University of New South Wales. A shotgun library of sequences of about 650 bp was prepared using the TruSeq DNA Sample Preparation kit (http://www.illumina.com/) and the library was sequenced on the MiSeq Sequencer (Illumina). The 250 bp paired-end sequences were assembled using CLC Genomics Workbench 6 (www.clcbio.com) and ABySS [23] using parameters as described in [24]. The mt contig of Ps2 was identified from the assembled contigs by BLASTn against GenBank nucleotide database, by the comparison of its contig length with published mt genomes of *T*. *indica* and *T*. *walkeri* and its very high sequencing depth due to high copy numbers of mt DNA. The mt contig obtained from each of the two assemblies was identical, and this was aligned with the reference mt genomes of *T*. *indica* (GenBank DQ993184) and *T*. *walkeri* (EF536375) using the ‘very accurate’ option of the alignment program in CLC Genomics Workshop 6 with default parameters of Gap open cost = 10 and Gap extension cost = 1.0. The alignment program also annotated the genes and coding sequences of the mt contig. The assembled and annotated mt genome of Ps2 was submitted to GenBank (KX394364).

### Analysis of PAVs in mt genomes of *T*. *indica*

**Fig 1:**
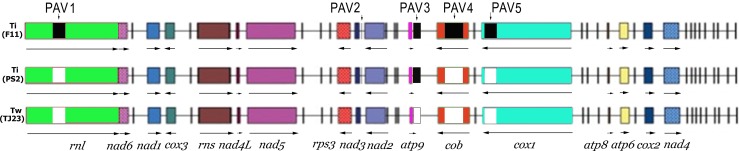
Alignment of the mt genomes of *T*. *indica* isolates, Ps2 (KX394364), F11 (DQ993184) and *T*. *walkeri* isolate, TJ23 (EF536375) showed identical gene order. Gene sizes are drawn to relative lengths and the arrows indicate the direction of transcription. The reverse arrow indicates transcription from the complementary strand. The black shaded and white unshaded boxes indicate presence and absence respectively of the corresponding presence/absence variation (PAV), labelled PAV1, PAV2, PAV3, PAV4 and PAV5 in the genomes.

The alignment of the mt sequences of the *T*. *indica* isolates, Ps2 (GenBank KX394364) and F11 (DQ993184), and the *T*. *walkeri* isolate, TJ23 (EF536375) indicated the positions of the five PAVs (Fig 1). Five sets of primers (Table 2) were designed for the analysis of the distribution of the five PAVs (Fig 1) in the collection of *T*. *indica* isolates.

**Table 2:** Sequences of primer pairs for the amplification of the five PAV elements; PAV1, PAV2, PAV3, PAV4 and PAV5 (Fig 1).

Primer Pairs (Sequence 5’-3’)	Nt position on ref seq, DQ993184	Annealing Temperature (AT) for temperature profile in PCR	Size(bp) With insert	Size (bp) Without insert
PAV1-For: TGGAGGATGAGATGAGTC	7934..7951	50°C	1289	51
PAV1-Rev: CATATTGTCCAAACGGTTTG	9222..9203			
PAV2-For: CTAATTCTTTTACCTGAGGTGC	35072..35093	50°C	370	149
PAV2-Rev: AGTTACTAAGTGTATTTGATGTTC	35418..35441			
PAV3-For: CTAATGATGAGTTTCCTTCTAC	40624..40645	68°C for 30 s with the AT decreasing by 1°C/cycle to 60°C, and then 57°C for 26 cycles	1521	76
PAV3-Rev: GTGTTTTATGCATAATGTAGTTG	42122..42144			
PAV4-For: TTAACAGAGAATCCACCC	43690–43707	65°C for 30 s with the AT decreasing by 1°C/cycle to 57°C, and then 57°C for 26 cycles	1715	69
PAV4-Rev: TCTTCTAAGTGCTATTCCTT	45404–45385			
PAV5-For: GAAAGCGTCTGGGTAATC	47365–47382	68°C for 30 s with the AT decreasing by 1°C/cycle to 60°C, and then 57°C for 26 cycles	1232	67
PAV5-Rev: TTTCCCTCAACATTTCCTAG	48596–48577			

Each PCR reaction was performed in a 10 μL volume of 2 mM MgCl_2_, 0.2 mM of each of the four deoxynucleotides (dATP, dTTP, dCTP and dTTP); 5 pmol of each of a primer pair, ~10 ng of genomic DNA and 1 unit of Taq DNA polymerase (Thermo Fisher, Scientific) in a buffer (50 mM Tris, pH 9.0, 20 mM NaCl, 1% Trition X-100, 0.1% gelatine). The PCR profile was an initial denaturation cycle of 94°C for 3 min; 35 cycles of 94°C for 30 s (denaturation), various annealing temperatures (primer pairs-specific-see Table 2) for 30 s, 72°C for 45 s (extension); and a final extension step of 72°C for 10 min.

Phylogenetic analysis of the ORFs in the PAVs was performed using the maximum likelihood criterion in the program, MEGA6 [25]. The data file comprises amino acid sequences of the ORFs for PAV1, PAV3, PAV4 and PAV5 and the protein IDs listed (S1 Table).

### LAMP assay

**Fig 2:**
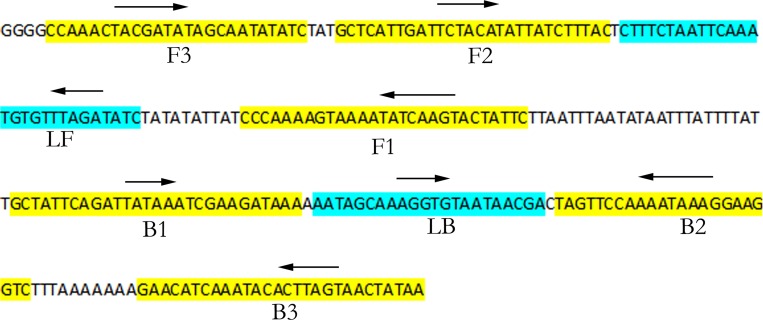
The mt segment, (KX394364: 33962..34226) encompasses a unique sequence of *T*. *indica* (PAV2). This is used for the design of primers (Table 3) in the LAMP assay. The primer sequences of the two outer primers (F3 and B3) and the two inner primers (FIP, BIP) are highlighted in yellow and the two loop primers are highlighted in blue. The orientations of the primer sequences in the assay are as indicated.

Four primers (Fig 2, Table 3), two inner primers (FIP and BIP) and two outer primers (F3 and B3) were designed to recognise a total of 6 separate regions in the target sequence (Fig 2) based on the principle in Notomi et al. [26]. Two additional loop primers (Fig 2, Table 3) were designed to anneal at the loop structure in LAMP amplicons to accelerate and enhance the sensitivity of the LAMP reaction.

**Table 3:** Sequences of primers for the LAMP assay of *T*. *indica*.

Primer name	Type	Sequence (5’-3’)	Length	%GC
*Ti*-F3	F3	CCAAACTACGATATAGCAATATATC	25	32
*Ti*-B3	B3	TTATAGTTACTAAGTGTATTTGATGTTC	28	25
*Ti*-FIP	FIP (*F1c + *F2)	GAATAGTACTTGATATTTTACTTTTGGG-GCTCATTGATTCTACATATTATCTTTAC	56	28.6
*Ti*-BIP	BIP (*B1c + *B2)	GCTATTCAGATTATAAATCGAAGATAAA-GACCTTCCTTTATTTTGGAACTA	51	29.5
*Ti*-LF	LF	GATATCTAAACACATTTGAATTAGAAAG	28	25
*Ti*-LB	LB	AATAGCAAAGGTGTAATAACGA	22	31.9

* Refer Fig 2.

A LAMP assay mix of a 25 μl volume comprised a mix of the 3 pairs of primers, F3/B3, FIP/BIP and LF/LB primer pairs in a final concentration of 0.2 μM, 1.6 μM and 0.4 μM respectively, 6 mM MgSO_4_, 1.4 mM each of the dNTP, 1 μl of the enzyme, Bst DNA Polymerase, Large Fragment (8000 U/ml) in 1X ThermoPol Buffer (New England Biolabs). The assay mix was incubated at 64°C for 30 minutes. One μl of a 6X gel loading buffer [30% (v/v) glycerol, 0.25%(w/v) bromophenol blue and 0.25% (w/v) xylene cyanol FF] containing a final concentration of 500X of the GelRed nucleic acid stain (http://www.biotium.com) was added to 5 μl of the LAMP products and loaded on a 2% agarose gel for electrophoresis in 1X TBE buffer at 90 V for 70 min. The separated products were visualized with the GelDoc XR+ System (Bio-Rad).

## Results and Discussions

### *T*. *indica* mt genome

The mt genome contig of Ps2 (KX394364) is 61,110 bp and has a sequencing depth of 5245x. The deep coverage is attributed to multiple copy numbers of mitochondria in fungal cells. The number of mitochondria/cell in budding yeast was estimated between 20 and 30 [27] and the number of mt DNA molecules per mitochondrion was reported in the range of 20–50 copies [28]. It was assumed that fungal mt genomes are circular, but experimental evidence had suggested that linear forms, possibly linear concatemers may be more common [29].

The alignment of the mt genome contig of Ps2 with the reference mt genome of *T*. *indica* and *T*. *walkeri* showed that the mt genomes of the two species have identical gene order (Fig 1). The mt genes annotated include 14 essential protein-coding genes (*atp6*, *atp8*, *atp9*, *cob*, *cox1-3*, *nad1-6 and nad4L*) for protein subunits of the mt complexes I, III, IV and V required for electron transfer and oxidative phosphorylation; *rps3*, which encodes the small ribosomal subunit protein S3, required for ribosome assembly; the small (*rns*) and large (*rnl*) subunit mt *rRNA*s and a set of 24 *tRNA* genes that is sufficient to translate the mt DNA-encoded proteome.

### Distribution of InDels and SNPs in the mt genomes

Genomes comparison has found two InDels of 30 nt (KX394364: 22193..22222) and 61 nt (KX394364: 56653..56713) present in both *T*. *indica* isolates (F11 and Ps2) and absent in *T*. *walkeri*, TJ23. There are seven short InDels which range from one to six nt, involving a total of fourteen nt, found between the two *T*. *indica* isolates (S2 Table). In contrast, there are 57 (involving a total of 132 nt) and 60 (involving a total of 142 nt) short InDels, which range from one to thirteen nt, between *T*. *walkeri* (TJ23) and the two *T*. *indica* isolates, F11 and Ps2 respectively (S2 Table). All these InDels are located in the small and large subunit *rRNA* genes and the non-coding regions only (S2 Table). No InDel had been found in the coding regions of protein and *tRNA* genes of both *Tilletia* species.

The numbers of SNPs in the aligned regions between the mt genomes of *T*. *walkeri* (TJ23) and the two *T*. *indica* isolates, F11 and Ps2, are 325 and 328 respectively, and this number is approximately nine times the number of SNPs (35) between the two *T*. *indica* isolates (Table 4). The number of SNPs between *T*. *indica* and *T*. *walkeri* represents only 0.5% of the aligned regions, and they are not evenly distributed. The proportions of coding regions in the mt genome of F11, Ps2 and TJ23 are 38%, 40% and 42% respectively. Fifty five percent (181/328) of the SNPs between the mt DNA of *T*. *indica* Ps2 and *T*. *walkeri* TJ23 are located in the coding regions. In contrast, 37% (11/35) of the SNPs between the mt genomes of the two *T*. *indica* isolates are located in the coding regions.

**Table 4:** Distribution of SNPs in mt genomes between a *T*. *indica* (Ps2) and a *T*. *walkeri* (TJ23) isolate, and between two *T*. *indica* isolates, F11 and Ps2.

	SNPs between *T*. *indica* (Ps2) and *T*. *walkeri* (TJ23)			SNPs between *T*. *indica* (Ps2) and *T*. *indica* (F11)		
Genetic Region	No amino acid substitution	Amino acid substitution	Total	No amino acid substitution	Amino acid substitution	Total
*rnl*	-	-	59	-	-	1
*rns*	-	-	74	-	-	11
*atp6*	2	0	2	0	0	0
*atp9*	4	0	4	0	0	0
*cox1*	8	0	8	1	0	1
*cox1* (intron between exon 1 and 2)	-	-	3	-	-	0
*cox1* (intron between exon 2 and 3)	-	-	3	-	-	1
*cox1* (intron between exon 3 and 4)	-	-	6	-	-	2
*cox1* (intron between exon 4 and 5)	-	-	18	-	-	3
*cox3*	4	0	4	0	0	0
*cob*	4	1	5	0	0	0
*nad1*	5	0	5	0	0	0
*nad2*	4	4	8	0	0	0
*nad3*	2	0	2	0	0	0
*nad4*	3	0	3	0	0	0
*nad5*	2	3	5	0	0	0
*nad5*(intron)	0	0	19	0	0	0
*nad6*	2	0	2	0	0	0
Non-coding regions	-	-	98	-	-	16
Total	40	8	328	1	0	35

The highest percentages of SNPs in the genes of mt genomes of *T*. *indica* (Ps2) and *T*. *walkeri* (TJ23) are found in the *rnl* and *rns* genes with proportions of ~18% and ~23% respectively (Table 4). There are 48 SNPs (~14.6%) located in the exons of protein-coding genes (Table 4), and eight resulted in amino acid substitutions in the genes; *cob*, *nad2* and *nad5*. A similar number of 49 SNPs (Table 4) are located in the introns of protein genes, *cox1* and *nad5*.

Comparison of SNPs in the two mt genomes of *T*. *indica* isolates, Ps2 and F11, also found the highest number in the non-coding regions (16/35 or ~46%) and in the *rns* gene (31%, Table 4). There is only one SNP in the *rnl* gene. The other SNPs occur only in the *cox1* gene, with 6 in the introns and one in the exon with no amino acid change (Table 4). No SNP has been found in the other protein coding genes.

The results suggested that about 85% of the SNPs that define sequence divergence between *T*. *indica* and *T*. *walkeri* mt genomes are located in the *rnl* and *rns* genes, the non-coding regions and the introns. A higher percentage of 97% of the SNPs between the 2 isolates of *T*. *indica* are located in these regions.

### PAVs in the mt genomes of *T*. *indica* isolates

**Fig 3:**
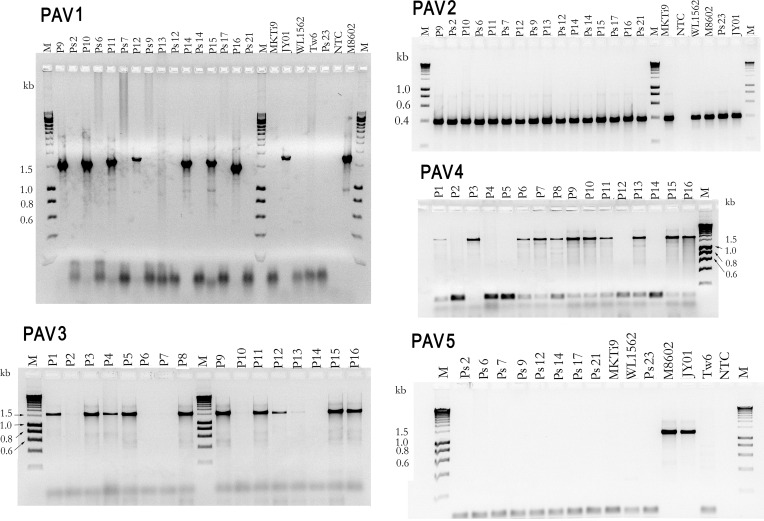
Distribution of PAVs in *T*. *indica* isolates. Amplification of PAV elements, PAV1 to PAV5 (Fig 1) using primers (Table 2) designed from analysis of the mt genomes alignment. The lengths of the amplicons with the PAV elements; PAV1, PAV2, PAV3, PAV4 and PAV5 are 1289, 370, 1521, 1715 and 1232 nt respectively (Table 2). NTC refers to no template control. *T*. *walkeri* isolate, Tw6, is a replicate of Tw4 (Table 1).

The 2 isolates of *T*. *indica*, F11 and Ps2, have different mt genome sizes of 65,147 nt and 61,110 nt respectively. Both are larger than *T*. *walkeri*, TJ23, which is 59,352 nt. There are five presence/absence variations (PAVs) in the mt genome of *T*. *indica* isolate, F11, which are absent in *T*. *walkeri* isolate, TJ23 (Fig 1), and they account for the much smaller mt genome of *T*. *walkeri*. PCRs across these five PAVs have found that they are also absent in *T*. *walkeri* isolates; DAR16720, DAR16802 and Tw4 (see e.g. in Fig 3 for PAV1 and PAV5).

Only two (PAV2 and PAV3) of the five PAVs in F11 are present in the *T*. *indica* isolate, Ps2 (Fig 1), and this explains the smaller mt genome size of Ps2. This result led to the design of 5 pairs of primers (Table 2) to study the distribution of these PAVs in a collection of *T*. *indica* isolates (Table 1).

Amplification with each of the 5 pairs of primers (Table 2) for a collection of *T*. *indica* isolates (Table 1) has enabled the determination of the distribution of the five PAVs in *T*. *indica* (Table 5). Only PAV2 (DQ993184: 35181..35401) is present in every *T*. *indica* isolate analyzed (Fig 3, Table 5). The other four PAVs; PAV1, PAV3, PAV4 and PAV5 are not present in every *T*. *indica* isolate analyzed (Fig 3). Analysis of their presence and absence in the collection of *T*. *indica* isolates separated them into 11 subgroups (Table 5). The two biggest sub-groups have the PAV profiles of ‘11111’ and ‘01100’ (Table 5) where ‘1’ and ‘0’ indicates ‘presence’ and ‘absence’ respectively and the position of the digit refers to the PAV of that position number, where position 1 refers to PAV1 and so forth. The PAV profile of isolate, Ps2, is ‘01100’ (Table 5) and is in agreement with the sequencing data.

**Table 5:** Profiles of the five PAVs in *T*. *indica* isolates.

Profiles	Isolates (Refer to Table 1)	Number
11111	Ti1, Ti3, Ti7, Ti9, Ti10, JyO1, P3, P9, P11, P15, P16,	11
11110	Ti2, P1, P8	3
01110	Ti6, P13,	2
11100	Ti8	1
01000	WL1562, Ps12	2
11010	P10	1
11001	P2, P14	2
01010	P6, P7	2
11011	M8602	1
01100	P4, P5, Ps2, Ps6, Ps7, Ps9, Ps14, Ps17, Ps21, Ps23	10
11101	P12	1
	Total	36

Profiles of presence/absence variations, PAV1, PAV2, PAV3, PAV4 and PAV5 (Refer Fig 1) represented in a 5-digit binary format where the first digit refers to PAV1, the second digit to PAV2 and so forth, and ‘1’ and ‘0’ indicates presence and absence respectively.

**Fig 4:**
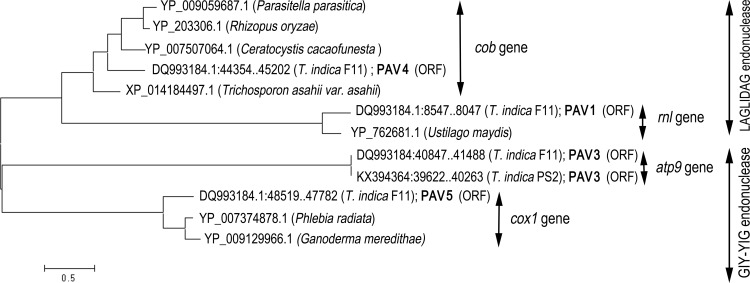
An unrooted phylogenetic tree generated by the maximum likelihood algorithm on a data file of amino acid/protein sequences of intron-encoded homing endonuclease genes (HEGs; LAGLIDAG and GIY-YIG endonucleases). Analysis used the Jones Taylor Thornton model with uniform substitution rates in the program MEGA6 [25]. Each of the four clusters of the intron sequences containing these HEGs are inserted in the same position in the mt gene indicated. The analysis demonstrated that the HEGs in PAV1, PAV4 and PAV5 of *T*. *indica* are more closely related with intron-encoded HEGs inserted at the same site in the same gene from diverse fungal species than the phylogeny of the fungal species, and is evidence of the horizontal transfer of these mobile introns, across taxonomic boundaries in fungi.

Four PAVs (1, 3, 4 and 5) are more than 1 kb long. PAV 1 and PAV 4 contain ORF with significant homology to the LAGLIDAG endonuclease, whilst PAV3 and PAV5 have ORF with significant homology to the GIY-YIG homing endonuclease family (Fig 4, S1 Table).

The LAGLIDADG endonuclease and GIY-YIG endonuclease are representative proteins of homing endonuclease genes (HEGs) in group 1 and group II introns [30, 31]. These HEGs confer intron mobility [30] and explain the apparent fluid distribution of these PAVs in *T*. *indica* isolates (Table 5). Group I and II introns possess highly site-specific recognition of intron-less alleles and can insert a copy of the intron in the allele via different homing mechanisms [30, 32].

PAV1 is located as an intron (GenBank DQ993184: 7955..9192) in the *rnl* gene (Fig 1). The insertion site has been found to be the same position (S3 Table) as a group 1 intron in the *rnl* gene in the mt genome of five *Ustilago maydis* strains; SRX3 (EU921808), SRX1(EU921806), MF14 (EU921802), BUB7 (EU921801) and FB1 (EU921800). This group 1 intron in *U*. *maydis* varies in size at 1126 and 1118 bp, depending on the strain (S3 Table). The InDels that resulted in length polymorphism of the group 1 intron are located outside the ORFs of the LAGLIDADG endonuclease encoded in the intron (S3 Table) and hence do not disrupt the coding sequence.

Our sequencing and PCR have confirmed that PAV1 is absent in isolate, Ps2 (KX394364, Table 5). PCRs have also found that PAV1 is absent in 16 out of 36 *T*. *indica* isolates screened (Table 5). We similarly found that this group I intron is absent in 4 out of 9 mt sequences of *U*. *maydis* isolates in the GenBank (S3 Table).

PAV4 (DQ993184.1: 43715..45360) is located as an intron in the *cob* gene (Fig 1) and possesses ORF for LAGLIDADG endonuclease (DQ993184.1: 44354..45202) with significant homology (2e-65 to 1e-70) to intron-encoded LAGLIDADG endonuclease in the same gene of mt genomes of fungi represented in Basidiomycota, Ascomycota and Mucuromycotina (S1 Table). These introns are inserted at the same site in the *cob* gene for the different fungal species (S1 Table).

PAV3 (DQ993184: 40660..42104, KX394364: 39434..40879) is of length 1,445 or 1446 bp and is located just adjacent to *atp9* in the intervening sequence between *atp9* and *cob* (Fig 1). It has an ORF (DQ993184: 40847..41488, KX394364: 39622..40263) with some similarity (7E-20 to 9E-33) to the GIY-YIG endonucleases in the basidiomycetous mt genomes of *Rhodotorula taiwanensis* RS1 (HF558455.1) and *Microbotryum lychnidis-dioicae* (NC_020353.1), which are also located in close proximity to *atp9* (S1 Table) but not at the same insertion site. The single InDel that results in length polymorphism in PAV3 in the two *T*. *indica* isolates, F11 and Ps2, is located outside the ORF and thus does not disrupt the ORF for the endonuclease (S1 Table).

PAV5 (DQ993184.1: 47402..48566) is located as an intron in the *cox1* gene (Fig 1) and has an ORF (DQ993184.1: 48519..47782) for a putative GIY-YIG type homing endonuclease with significant homology (4e-81 to 2e-88) to the same endonuclease family in a group I intron in the *cox1* gene in basidiomycetous mt genomes (S1 Table) of *Phlebia radiate* (NC_020148.1) and *Ganoderma meredithae* (NC_026782). These group 1 introns in different fungal species have the same insertion site (S1 Table) in the *cox1* gene.

Group 1 introns inserted at the same site of a gene have been reported to be evolutionary related [33]. The GIY-YIG endonuclease encoded in PAV3 did not have the same insertion site as the similar endonuclease in the vicinity of *atp9* for *Rhodotorula taiwanensis* RS1 and *Microbotryum lychnidis-dioicae* (S1 Table) and this is reflected in the much reduced statistical significance in the similarities of their sequences (S1 Table). This study has demonstrated that the intron-encoded HEGs inserted at the same site in the same mt gene of widely diverse fungal species (S1 Table) are more closely related than the fungal species themselves (Fig 4), and is evidence of the occurrence of horizontal transfer of mobile introns in fungal mt genomes across taxonomic boundaries in fungi (Fig 4). Such horizontal transfer has also been reported for group 1 introns in *Gaeumannomyces graminis* [33], Sclerotiniaceae [34] and *Glomus* species [35].

### Basis of a LAMP assay for *T*. *indica*

PAV2 (DQ993184: 35181..35401, KX394364: 33956..34176) is 221 bp and is present in every *T*. *indica* isolate screened (36 in Table 1 and 10 unpublished), and is absent in its closest relative, *T*. *walkeri*. It is situated in the intervening sequence between the genes for *tRNA-Trp* and *NAD3* (Fig 1). BLASTn analysis has indicated that this DNA sequence is unique and specific to *T*. *indica* with no homology to any other fungi or microorganism. PAV2 has no ORF and is AT rich (75%). No SNP has been found in this region. Its biological significance is unknown but it is a unique genetic fingerprint of *T*. *indica*. This study has thus used this genetic region to design a successful LAMP assay for *T*. *indica* (see ‘LAMP assay’ under ‘Methods‘ above). The target sequence in the LAMP assay extends slightly on both ends to give a target length of 265 bp (KX394364: 33962..34226, Fig 2).

**Fig 5:**
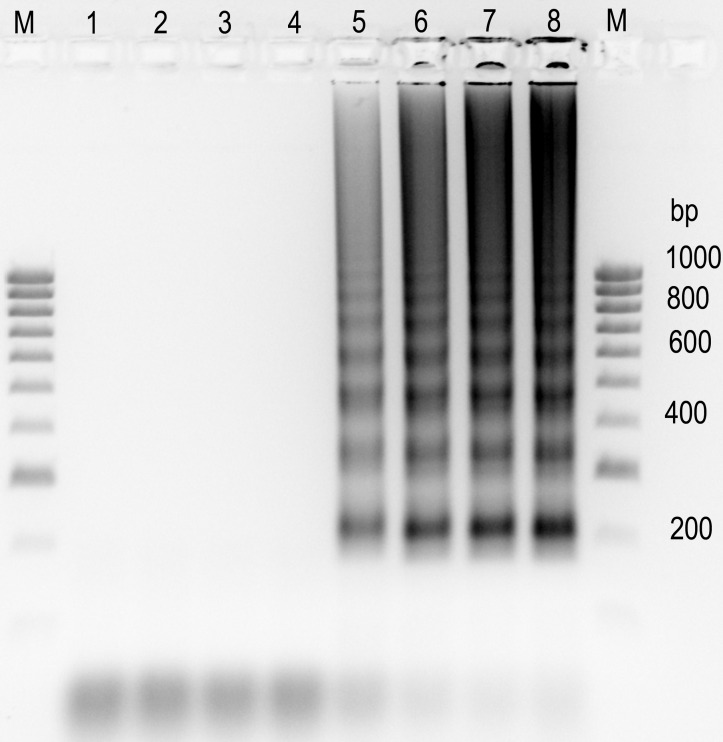
Detection of LAMP amplicons. A positive LAMP reaction can be visualized as a ladder of DNA loop amplicons on an agarose gel using the nucleic acid fluorescent stain, GelRed. Sensitivity of the LAMP assay was determined at approximately 10 pg (lane 5) using a 1 in 10 DNA dilution series from 10 ng (lane 8) to 0.01 pg (lane 2). Lane 1 is no template control.

The LAMP products were analyzed on an agarose gel and positive reactions were visualized as ladder-like patterns using a fluorescent stain, GelRED (Fig 5). A positive reaction can also be visualized by the formation of a white precipitate that makes the solution turbid [36]. The amount of turbidity correlates with the amount of DNA. Analysis with isolates of other *Tilletia* species including *T*. *walkeri*, *T*. *ehrhartae*, *T*. *tritici* (*T*. *caries*), *T*. *laevis*, *T*. *fusca*, *T*. *contraversa*, *T*. *bromi* and *T*. *horrida* (Table 1) gave negative results, confirming the LAMP assay developed is specific to *T*. *indica* only.

**Fig 6:**
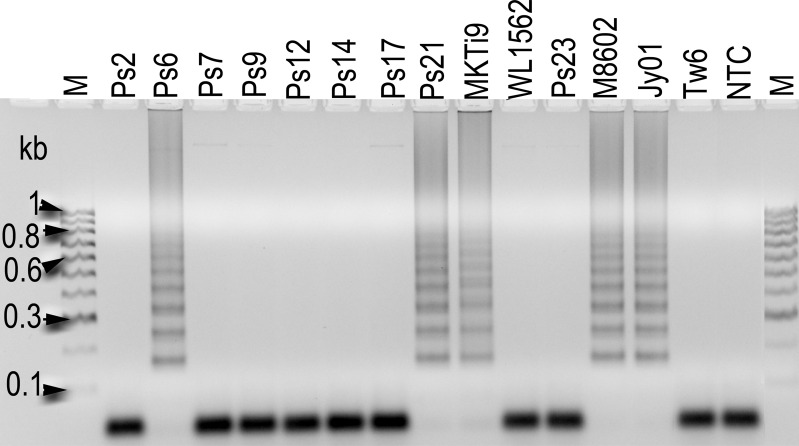
False negative results from LAMP assay by Gao et al. [37]. A positive reaction is indicated as a ladder of DNA fragments on an agarose gel. Eight of thirteen *T*. *indica* isolates were tested ‘negative’ as predicted by the absence of PAV5 (Fig 3). The positive results obtained for isolates Ps6, Ps21 and MKTi9 with assay by [37] suggested the presence of PAV5 in a few copies of mt DNA in these isolates.

A recent publication of a LAMP assay for *T*. *indica* [37] used a target sequence that lies in the element, PAV5 and had screened only a very small set of five *T*. *indica* isolates. PCR analysis have indicated an ‘absence’ of PAV5 in sixty one per cent of the *T*. *indica* isolates screened in this study (Table 5, Fig 3). These isolates are thus predicted to give false negative results for the *T*. *indica* LAMP assay by Gao et al. [37]. This has been confirmed for *T*. *indica* isolates; Ps2, Ps7, Ps9, Ps12, Ps14, Ps17, WL1562 and Ps23 (Fig 6) which gave negative results for the assay by Gao et al. [37]. A false negative will result in serious, costly consequences for the industry. The LAMP assay developed by Gao et al. [37] is thus likely to be unreliable and should not be used for quarantine diagnosis.

Some *T*. *indica* isolates which have been found by PCR analysis to have no PAV5 (Fig 3) have tested positive (e.g. isolates Ps6, Ps21 and MKTi9 in Fig 6) in the LAMP assay by Gao et al. [37]. This observation suggested that PAV5 is present in a few copies of mt DNA in these isolates and was not detected during PCR for PAV5, as the ‘absent’ variant, present in higher copy numbers, was preferentially amplified. The element PAV5 appears to exist in a dynamic state in *T*. *indica* isolates. The ratio of mt DNA of (PAV5 absence) versus (PAV5 presence) in *T*. *indica* has been found to range from total absence (e.g. Ps2 and Ps7 in Figs 3 and 6) to total presence (e.g. M8602 and Jy01 in Figs 3 and 6) with some isolates having ratios in between (e.g. Ps6 and Ps21 in Figs 3 and 6).

The sensitivity of our assay was determined to be 10 pg of fungal DNA (Fig 5) and this corresponds to the sensitivity of LAMP assay reported by Rigano et al. [38]. A LAMP assay for *Fusarium graminearum* DNA has reported a higher sensitivity of ~2 pg [39]. Our assay developed is highly sensitive and specific and can be performed on a simple heating block, by general technical staff in a resource-limited laboratory, for example a quarantine station or a grain receiver laboratory. The simple procedure of the LAMP assay and the easy detection formats will enable the assay to be adapted into a robotic set-up and automation for high-throughput in an incursion.

## Conclusion

Comparative mt genome analysis of *T*. *indica* (KX394364 and DQ993184) and *T*. *walkeri* (EF536375) has found 325 to 328 SNPs, two InDels (30 and 61 nt), 57 to 60 short InDels (range from 1 to 13 nt and total 132 to 142 nt) and five PAVs between the two species. All five PAVs are absent in *T*. *walkeri*. There are four PAVs of size > 1 kb, which are not present in every *T*. *indica* isolates. Analysis of their presence and absence separated the *T*. *indica* isolates into 11 sub-groups. They are present as introns in mt genes. These introns possess ORF that codes for putative HEGs characteristic of mobile group I and II introns. The much smaller PAV2 of 221 bp, present in every *T*. *indica* isolate and absent in *T*. *walkeri*, was used as the genetic fingerprint for the successful development of the LAMP assay for *T*. *indica*. This LAMP assay should supersede the assay reported by Gao et al. [37] which has been shown to give some false negative results for *T*. *indica*, which will have potentially serious costly consequences for the industry (see ‘Results and Discussion‘).

## Supporting Information

S1 Table ORFs in PAVs. BLAST analysis of open reading frames (ORFs) in PAVs (> 1 kb) in *T*. *indica* mt sequences, DQ993184 and KX394364.
(PDF)

S2 Table Distribution and comparison of InDels in the mt genomes of *T*. *indica* and *T*. *walkeri*. (XLSX)

S3 Table PAV1 in the *rnl* gene. Group 1 intron with putative LAGLIDADG endonuclease at the same insertion site in the *rnl* gene in mt genomes of *T*. *indica* and *U*. *maydis*.
(PDF)
