# Supplementary material for: Characterization of SNP and Structural Variations in the Mitochondrial Genomes of Tilletia indica and Its Closely Related Species Formed Basis for a Simple Diagnostic Assay
Source: PLoS One. 2016 Nov 4;11(11):e0166086. doi: 10.1371/journal.pone.0166086 (PMC5096740; doi:10.1371/journal.pone.0166086)
Supplement: S1 Table — BLAST analysis of open reading frames (ORFs) in PAVs (> 1 kb) in T. indica mt sequences, DQ993184 and KX394364. (PDF) [file pone.0166086.s001.pdf]

S1 Table. ORFs in PAVs

Insertion element	Sequence/putative domain description	Seq/Protein ID	Expect	Phylum	comment
DQ993184:7955-9192 (**PAV 1**), ORF: DQ993184.1:8547..8047	LAGLIDADG endonuclease (mitochondrion) [*Ustilago maydis*]	YP_762681.1	6E-56	Basidiomycota	group 1 intron in *rnl* **same insertion site**
	Sporisorium reilianum SRZ2 mt DNA sequence; FQ311469:75578..76831	Not annotated	NA	Basidiomycota	Intron in *rnl* **same insertion site**
DQ993184:40660..42104 (**PAV 3**), ORF: DQ993184:40847..41488; KX394364: 39434..40879, ORF: KX394364: 39622..40263	GIY-YIG endonuclease (mitochondrion) [*Rhodotorula taiwanensis* RS1]	CCO62245.1	9.00E-33	Basidiomycota	[a]CDS in intron in *atp9* **different insertion site**
	hypothetical protein (mitochondrion) [*Microbotryum lychnidis-dioicae*]	YP_007475398.1	7.00E-20	Basidiomycota	CDS adjacent to *atp9* **different insertion site**
DQ993184.1:43715..45360 (**PAV 4**), ORF: DQ993184.1:45202.. 44354	LAGLIDADG/HNH endonuclease (mitochondrion) [*Parasitella parasitica*]	YP_009059687.1	1.00E-70	Mucoromycotina	intron in *cob* **same insertion site**
	orf291 (mitochondrion) [*Rhizopus oryzae*]	YP_203306.1	2.00E-68	Mucoromycotina	intron in *cob* **same insertion site**
	Bi3p (mitochondrion) [*Trichosporon asahii var. asahii* CBS 2479]	XP_014184497.1	3E-66	Basidiomycota	Not annotated
	LAGLIDADG endonuclease (mitochondrion) [*Ceratocystis cacaofunesta*]	YP_007507064.1	2E-65	Ascomycota	intron in *cob* **same insertion site**
DQ993184.1:47402..48566 (**PAV 5**), ORF: DQ993184.1:48519..47782	hypothetical protein PRA_mt0037 (mitochondrion) [*Phlebia radiata*]	YP_007374878.1	2E-88	Basidiomycota	Group 1intron in *cox1* **same insertion site**
	GIY-YIG type homing endonuclease (mitochondrion) [*Ganoderma meredithae*]	YP_009129966.1	4E-81	Basidiomycota	Group 1 intron in *cox1* **same insertion site**

BLAST analysis of open reading frames (ORFs) in PAVs (> 1 kb) in *T. indica* mitochondrial sequences, DQ993184 and KX394364.
