# Supplementary material for: Characterization of SNP and Structural Variations in the Mitochondrial Genomes of Tilletia indica and Its Closely Related Species Formed Basis for a Simple Diagnostic Assay
Source: PLoS One. 2016 Nov 4;11(11):e0166086. doi: 10.1371/journal.pone.0166086 (PMC5096740; doi:10.1371/journal.pone.0166086)
Supplement: S3 Table — Group 1 intron with putative LAGLIDADG endonuclease at the same insertion site in the rnl gene in mt genomes of T. indica and U. maydis. (PDF) [file pone.0166086.s003.pdf]

S3 Table. PAV1 in the *rnl* gene

Seq ID	PAV1/intron location	ORF (LAGLIDADG endonuclease)	Species and strain	Insert size
DQ993184	7955-9192	8547..8047	*T. indica* F11	1238
KX394364	No insertion	NA	*T. indica* PS2	0
EU921808	6929..5804	6391..6801	*U. maydis* SRX3	1126
EU921807	No insertion	na	*U. maydis* SRX2	0
EU921806	8298..7173	7760..8170	*U. maydis* SRX1	1126
EU921805	No insertion	NA	*U. maydis* GF5	0
EU921804	No insertion	NA	*U. maydis* MF34	0
EU921803	No insertion	NA	*U. maydis* MF18	0
EU921802	6339..5214	5801..6211	*U. maydis* MF14	1126
EU921801	8285..7168	7747..8157	*U. maydis* BUB7	1118
EU921800	6331..5214	5793..6203	*U. maydis* FB1	1118

Group 1 intron with putative LAGLIDADG endonuclease at the same insertion site in *rnl* gene in mt genomes of *T. indica* and *U. maydis.*
